# Time-Course Changes and Role of Autophagy in Primary Spinal Motor Neurons Subjected to Oxygen-Glucose Deprivation: Insights Into Autophagy Changes in a Cellular Model of Spinal Cord Ischemia

**DOI:** 10.3389/fncel.2020.00038

**Published:** 2020-03-20

**Authors:** Shudong Chen, Ruimin Tian, Dan Luo, Zhifeng Xiao, Hui Li, Dingkun Lin

**Affiliations:** ^1^The Second Affiliated Hospital of Guangzhou University of Chinese Medicine, Guangzhou, China; ^2^Guangdong Provincial Academy of Chinese Medical Sciences, Guangzhou, China; ^3^School of Basic Medical Sciences, Guangzhou University of Chinese Medicine, Guangzhou, China

**Keywords:** spinal motor neurons (SMNs), spinal cord ischemia, cellular model, oxygen-glucose deprivation (OGD), autophagy

## Abstract

Spinal cord ischemia is a severe clinical complication induced by thoracoabdominal aortic surgery, severe trauma, or compression to the spinal column. As one of the most important functional cells in the spinal cord, spinal motor neurons (SMNs) suffer most during the process since they are vulnerable to ischemic injury due to high demands of energy. Previous researches have tried various animal models or organotypic tissue experiments to mimic the process and get to know the pathogenesis and mechanism. However, little work has been performed on the cellular model of spinal cord ischemia, which has been hampered by the inability to obtain a sufficient number of pure primary SMNs for *in vitro* study. By optimizing the isolation and culture of SMNs, our laboratory has developed an improved culture system of primary SMNs, which allows cellular models and thus mechanism studies. In the present study, by establishing an *in vitro* model of spinal cord ischemia, we intended to observe the dynamic time-course changes of SMNs and investigate the role of autophagy in SMNs during the process. It was found that oxygen-glucose deprivation (OGD) resulted in destruction of neural networks and decreased cell viability of primary SMNs, and the severity increased with the prolonging of the OGD time. The OGD treatment enhanced autophagy, which reached a peak at 5 h. Further investigation demonstrated that inhibition of autophagy exacerbated the injury, evidencing that autophagy plays a protective role during the process.

## Introduction

Spinal cord ischemia is a devastating complication following thoracoabdominal aortic surgery (Drinkwater et al., 2010; Shimizu and Yozu, 2011; Etz et al., 2014), and it frequently occurs as well when the spinal cord suffers from direct trauma during spinal cord injury (Rivlin and Tator, 1978; Aslan et al., 2009), or compression from vertebral stenosis and various other spinal lesions (Gooding et al., 1975; Griffiths et al., 1979; Yang et al., 2015), which may lead to various degrees of disability or even paraparesis/paraplegia (Coselli et al., 1997; Etz et al., 2008; Suarez et al., 2013). During the spinal cord ischemia, the blood flow of the spinal cord is reduced, and the tissue auto-regulation is disrupted (Kise et al., 2015; Weidauer et al., 2015), which takes an essential part in the pathophysiological mechanisms. During the past decades, numerous studies, by adopting various animal models (in rats, mice, rabbits, cats, pigs, and primates; Griffiths et al., 1979; Kato et al., 1997; Kolenda et al., 2003; Wang et al., 2010; Hwang et al., 2012, 2017; Nazli et al., 2015; Yang et al., 2015) and tissue (organotypic spinal cord slices) experiments (Turner and Johnson, 2011; Esposito et al., 2012) of spinal cord ischemia, have been studied for the pathogenesis and developed some methods to reduce the injury; however, up until now, the treatment options remain strongly controversial and limited, and the concrete mechanisms and roles of certain types of cells involved in this process are still far from clear. Besides *in vivo* or organotypic models, cellular models of a certain disease to mimic a certain cell type phenotype hold the key to understand the pathogenesis of a disease; nevertheless, progress in performing researches about spinal cord ischemia have been impeded by the inability to gain sufficient number with well uniformity of certain types of cells *in vitro*.

In eukaryotic cells, the maintenance of normal metabolic balance relies on two major protein catabolism pathways: the ubiquitin–proteasome pathway (UPS) and autophagy lysosomal pathway. A number of neurological disorders have been discovered to be associated with autophagy. Autophagy (means “self-eating” in Greek, “auto” oneself, “phagy” to eat), a term which was first coined by Christian de Duve in 1963 (De Duve, 1963; Klionsky, 2008), is a highly regulated process in which protein aggregates and damaged organelles are degraded *via* the lysosomal pathway. It is an evolutionary conserved catabolic system where unnecessary or dysfunctional cellular components, including cytosolic proteins and organelles, are detained in a double-membrane vesicle, and the resulting vacuoles (autophagosomes) are degraded after they are transmitted to the lysosomal compartment (Klionsky and Emr, 2000; Mizushima and Komatsu, 2011; Feng et al., 2014). It serves like a cellular housekeeper, to keep the amino acid/energy recycling and try to mitigate various metabolic stresses (Levine and Kroemer, 2008; Wirawan et al., 2012). Although some of abovementioned functions overlap with those of the UPS, autophagy mainly contributes to the turnover of long-lived proteins and the maintenance of amino acid pools in the setting of cellular stresses (Ciechanover et al., 2000; Nedelsky et al., 2008; Lu et al., 2013). This process is considered to be adaptive and essential for survival, differentiation, development, and homeostasis under both physiological and pathological conditions. In various neurological diseases, autophagy may be either upregulated or downregulated or even impaired (Komatsu et al., 2006; Lee et al., 2010; Lynch-Day et al., 2012; Gu et al., 2013; Martin et al., 2015). Previous *in vivo* studies have indicated that autophagy is implicated in the spinal cord ischemic injury (Kanno et al., 2009a; Fan et al., 2014; Fujita et al., 2015; Fang et al., 2016), whereas the concrete mechanisms during the process are controversial, and the impact of autophagy in primary spinal neurons has not been fully understood. Accumulating evidence suggested that autophagy may act as a “double-edged sword” with regard to central nervous system injury. The role of autophagy varies with the type or degree of injuries. Mounting studies showed that autophagy activation may be involved in neuroprotection in cerebral or spinal cord injury (Sheng et al., 2010; Mariño et al., 2011; Wang P. et al., 2012; Sun et al., 2016), but some investigators reported that the ischemic injury may induce “autophagic cell death,” and inhibition of autophagy can prevent neuron death after ischemic injury (Yu et al., 2004; Rami et al., 2008; Uchiyama et al., 2008; Kanno et al., 2009b). Some *in vivo* studies have also documented that, in the model of cerebral ischemia, autophagy is activated in various cell types—neurons, astrocytes, and vascular endothelial cells, while in the model of spinal cord injury (Kanno et al., 2009a; Fang et al., 2016), autophagesomes mainly accumulate in neurons, microglia, or oligodendrocytes, rather than in astrocytes. Nevertheless, the concrete change in a certain cell type during the process of spinal cord injury has never been reported, and the underlying mechanisms deserve further investigation.

Spinal motor neurons (SMNs), the nerve cells that connect the ventral horn of the spinal cord to directly or indirectly control muscles or glands, act as one of the most important neurons in the spinal cord. Existing studies have demonstrated that SMNs are vulnerable to ischemic injury due to their high demands of energy (Kanno et al., 2009a; Fujita et al., 2015; Fang et al., 2016). The ischemia induces changes in SMNs, which can, in turn, affect the process. Nevertheless, little is known about the dynamic time-course changes and the function of autophagy in SMNs during the process. By optimizing the isolation and culture of SMNs, our laboratory has developed an improved culture system of SMNs, which allows establishing cellular models and performing mechanism studies. Based on this culture system, we also tried some *in vitro* models of SMNs to mimic the metabolic perturbation occurring *in vivo* during the spinal cord ischemic injury, which may include the state of hypoxia, aglycemia, acidosis, oxidative stress, etc. Oxygen-glucose deprivation (OGD), which has been widely utilized to study the cerebral ischemia injury or ischemia/reperfusion injury, is a commonly used model to mimic an ischemic milieu *in vitro* (Fontella et al., 2005; Cimarosti et al., 2012; Wang R. et al., 2012; Tasca et al., 2015). Performing OGD-induced SMNs injury model can well mimic the extracellular condition in spinal ischemia and results in neuronal insult; however, unlike cerebral ischemia injury and ischemia/reperfusion injury, which have been studied by numerous researches, investigations about the spinal neurons are far from enough.

To date, no studies have addressed the time-course changes and the role of autophagy in primary SMNs subjected to OGD conditions. In the current study, by establishing a cellular model of spinal cord ischemia *in vitro*, we intended to investigate the potential involvement of autophagy and its time course in primary SMNs. Furthermore, by treating with the autophagy inhibitor, the role of autophagy during the process was clearly observed.

## Materials and Methods

### Reagents and Chemicals

Neurobasal^TM^ medium, B-27^TM^ supplement, HBSS, PBS, and trypsin were purchased from Gibco. Brain-derived neurotrophic factor (BDNF), glial cell-derived neurotrophic factor (GDNF), glutamate, L-glutamine, Dnase, poly-D-lysine (PDL), paraformaldehyde (PFA), and 4′,6-diamidino-2-phenylindole (DAPI) were all obtained from Sigma-Aldrich. RIPA Lysis and Extraction Buffer, as well as Pierce^TM^ BCA Protein Assay Kit were purchased from Thermo Scientific. The protease inhibitor cocktail was from Bimake. Primary antibodies against NGF receptor (p75^NTR^; ab6172), CHAT (ab6168), and β-actin (ab8226) were provided by Abcam. Antibody against SMI 32 (NE1023), Cy3-conjugated sheep anti-rabbit IgG secondary antibody (AC111C), and polyvinylidene difluoride (PVDF) membranes were purchased from EMD Millippore. Primary antibody against light chain 3 (LC3; M152-3) was purchased from MBL International. Primary antibodies against Beclin1 (#3738), SQSTM1/p62 (#5114), as well as horseradish peroxidase (HRP)-linked secondary antibodies (#7074, #7076) were obtained from Cell Signaling Technology. Alexa fluor 488-conjugated donkey anti-mouse IgG secondary antibody (A-21202) was bought from Invitrogen. Clarity^TM^ Western ECL Substrate was obtained from Bio-rad. CCK8 kit were purchased from Dojindo. Bafilomycin A1 (Baf-A1) and 3-Methyladenine (3-MA) were purchased from Selleck Chemicals.

### Isolation and Culture of Primary Spinal Motor Neurons (SMNs)

Pregnant Sprague–Dawley rats were used in the experiments in accordance with internationally accepted standard guidelines for animal use and care, and our study protocol was reviewed and approved by the ethical committee of Guangzhou University of Chinese Medicine. SMNs were prepared from 14- to 16-day embryonic rats as we have reported previously (Chen et al., 2018). Briefly, the spinal cords from embryonic rats were dissected, with vessels and menings gently removed. Then, tissues were washed and cut into small slices and transferred a new dish for trypsinization (0.25% trypsin and 0.4% Dnase) for 20 min at 37°C, followed by adding complete media to inactivate the trypsin, Later, the tissues were gently triturated using Gilson blue pipette tips and a 100-mesh filter to obtain the single-cell suspension. Cells were harvested by centrifugation, re-suspended by Neurobasal^TM^ medium supplemented with 2% B27, and plated on panning dishes, which had been coated with affinity-purified goat anti-mouse IgG in Tris-HCl buffer at 4°C overnight, washed, and then incubated with p75^NTR^ antibody (1–10 μg/ml in PBS). After immunopanning, panning dishes were gently washed to remove those loosely attached cells. Adherent cells (phase bright and quite big under the inverted phase-contrast microscope) were collected by trypsinization and centrifugation, then re-suspended by plating media, whose components were Neurobasal medium supplemented with 2% B27 and 25 μM Glutamate. An aliquot (2 μl) was used for counting before cells were seeded at a proper density (2 × 10^4^/ml–5 × 10^4^/ml) on Petri dishes or a 96-well plate, which had been pre-treated with PDL (20 μg/ml, for 30 min at room temperature). 24 hours after plating, media was fully replaced by growth media, whose components were Neurobasal medium supplemented with 2% B27, 2 mM of L-Glutamine, 10 ng/ml of BDNF, and 10 ng/ml of GDNF. Neurons were maintained at 37°C in a humidified 5% CO_2_ incubator, and half volume of the medium was changed with fresh media every 3 days. Experiments were performed after day *in vitro* (DIV) 7–14.

### Oxygen-Glucose Deprivation (OGD) Exposure of SMNs

**Figure 1:**
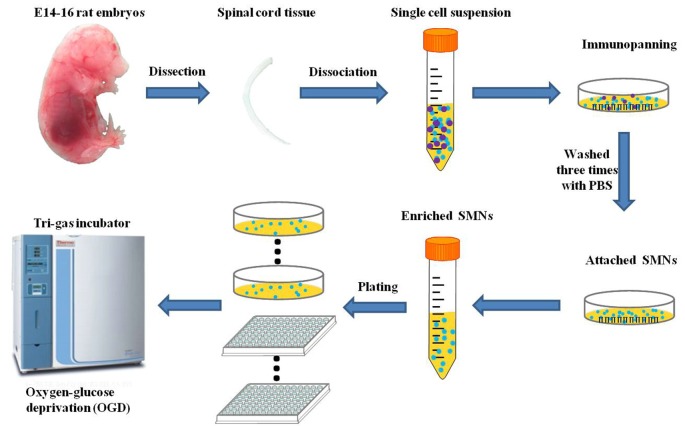
Schematic diagram of the isolation and purification of primary spinal motor neurons (SMNs) and oxygen-glucose deprivation (OGD) modeling process.

OGD has been utilized as a simulation model of hypoxic insult *in vitro*. OGD experiments were performed between DIV 7 and DIV 14, at which time SMNs represented at least 95% of the population as assessed by SMN marker and DAPI staining. To initiate OGD, the cultured primary SMNs were rinsed with HBSS and then incubated with Earle’s balanced salt solution (EBSS), whose components were (in mg/L): 6,800 NaCl, 400 KCl, 200 CaCl_2_, 200 MgSO_4_–7H_2_O, 140 NaH_2_PO_4_–H_2_O, 2,200 NaHCO_3_, pH 7.4, and placed in a tri-gas incubator (Thermo Fisher Scientific), which was set at 5% O_2_ and 5% CO_2_ at 37°C for certain hours to mimic ischemic insult. The experimental schedule is shown in Figure 1. Control cells were cultured in normal culture medium (Neurobasal medium supplemented with 2% B27 and 2 mM L-Glutamine) and placed in the regular incubator (95% air and 5% CO_2_ at 37°C).

### Quantification of Cell Viability With CCK8 Assay

Cell viability of cultured SMNs was tested with a nonradioactive cell counting kit (CCK8) following the manufacturer’s instructions. In brief, the primary SMNs were seeded on 96-well plates at a density of 2.5 × 10^3^ per well. After cells were subjected to OGD for 0.5, 1, 2, 3, 5, and 7 h, 10 μl of CCK8 was added to each well, and the mixture was incubated for 4 h at 37°C. Then, absorbance was determined at 450 nm by a microplate reader. Results were expressed as the percentage of CCK8 reduction, and the absorbance of control cells was set at 100%.

### Immunofluorescence Assay

Isolated SMNs were plated on confocal dishes for immunofluorescence assay. Cells were washed with ice-cold PBS, fixed with 4% paraformaldehyde solution for 15 min at room temperature, permeabilized by 0.2% Triton X-100 (in PBS, pH 7.4) and further blocked in 5% goat serum for 1 h at room temperature. Cells were then incubated with appropriate primary antibodies at 4°C overnight. For double immunofluorescence, different primary antibodies from different species were simultaneously incubated. For the identification of SMNs, mouse anti-SMI 32 (1:500) and/or rabbit anti-CHAT (1:500) were used. While for the identification of autophagic marker, mouse anti-LC3 (1:500) was used. After being washed, cells were incubated with Alexa fluor 488-conjugated donkey anti-mouse IgG (1:1,000) or/and Cy3 conjugated sheep anti-rabbit secondary antibody (1:1,000) for 1 h in dark. For nuclear counterstaining, DAPI was used. Labeled cells were identified using Olympus FX-70 fluorescence microscope or Zeiss LSM 510 META laser scanning confocal microscope, and digital images were recorded by Adobe Photoshop software and Zeiss LSM Image Examiner software.

### Protein Isolation, Quantification, and Western Blot Analysis

Isolated SMNs were plated on Petri dishes for Western blot analysis. After OGD treatment, cells were harvested and lysed in ice-cold RIPA lysis buffer with protease inhibitor cocktail. Protein concentrations were detected using BCA protein assay kit, and proteins in each group were adjusted to the same concentrations. Equivalent amounts of protein were loaded and electrophoretically on 12% or 15% sodium dodecyl sulfate polyacrylamide gel electrophoresis (SDS-PAGE), and blotted onto PVDF membranes. Then, the nonspecific binding was blocked by incubating membranes in 5% skimmed milk (in TBS containing 0.05% Tween 20) for 1 h. The membranes were incubated with primary antibodies diluted in TBST as follows: mouse anti-LC3 (1:1,000), rabbit anti-Beclin1 (1:1,000), rabbit anti-SQSTM1/p62 (1:1,000), and mouse anti-β-actin (1:3,000) overnight at 4°C. The next day, membranes were washed three times with TBST for 5 min each wash, followed by incubating with anti-mouse IgG HRP-linked antibody (1:2,000) or anti-rabbit IgG HRP-linked antibody (1:2,000) for 1 h at room temperature. Immunoreactivity was detected with Clarity^TM^ Western ECL Substrate, and reacting bands were captured by Bio-rad ChemiDoc^TM^ XRS+ system, and analyzed for final determination of protein expression with Image Lab and normalized by β-actin as internal controls.

### Electron Microscopy

The neurons were harvested and fixed in 2.5% glutaraldehyde in 0.1 M phosphate buffer (pH 7.4) for 8 h, followed by treatment with 1% osmium tetroxide for an additional 1 h, and dehydrated by gradient ethanol and acetone. Samples were immersed in resin, hardened, and sectioned (50–70 nm). Later, the ultrathin sections were stained with lead citrate and uranyl acetate and observed by electron microscopy (Zeiss EM910).

### Statistical Analysis

All experiments were performed at least three times independently. Statistical analysis of all data was performed using SPSS 22.0 and GraphPad Prism 7. Results were presented as mean ± standard deviation (SD). Statistical comparisons between two groups were determined using unpaired Student’s *t*-test. Differences among groups were evaluated with one-way analysis of variance (ANOVA) followed by Tukey’s or Dunnett’s *post hoc* test when appropriate, or two-way ANOVA followed by Sidak’s multiple comparisons test. A difference was considered statistically significant when *p* < 0.05.

## Results

### Characterization, Identification, and Purity of Primary SMNs

**Figure 2:**
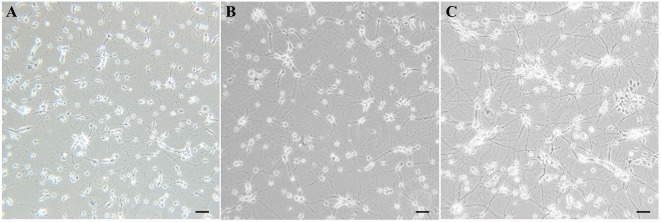
Visual characterization of cultured primary SMNs. **(A)** Morphology of cultured cells after plating for 2 days. **(B)** SMNs developed large cell bodies with long neurites. **(C)** Mature SMNs formed vastly interconnected neurite networks.

Within 24 h after plating, most cells were adherent to the bottom, and some even grew tiny neurites, but all cells were solitary. After the first media replacement (24 h) to remove the floating cells and change some media components, larger cells appeared, cell outgrowths grew longer and formed multiple branches. After 7 days in culture, cells grew mature with plump soma and high refraction, and formed interconnected neural networks diffusely (Figure 2).

**Figure 3:**
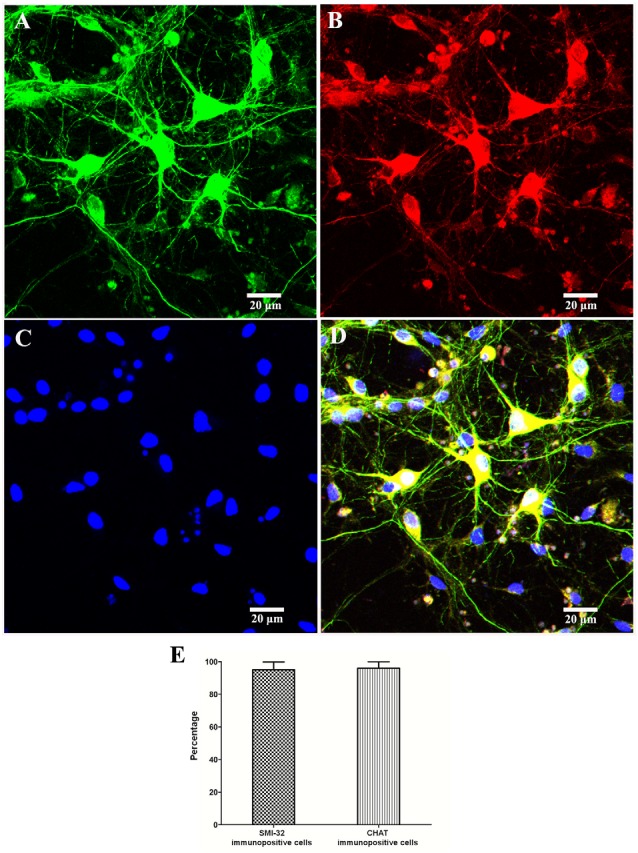
Identification and purity of SMNs by immunofluorescence with motor neuron-specific markers against SMI-32 and CHAT. The immunofluorescence assay of motor neuron marker SMI-32 (green, **A**) and CHAT (red, **B**) showed cell bodies, dendrites, and large axons, with 4′,6-diamidino-2-phenylindole (DAPI) staining (blue, **C**) showing the nuclei. **(D)** Merged image. **(E)** The percentage of SMI-32 or CHAT-immuopositive cells were calculated. Data were presented as mean ± SD (*n* = 5 independent experiments). At least 10 random fields from one sample were averaged in each independent experiment. Scale bars represent 20 μm.

In order to identify motor neurons in the culture system, SMI-32 and CHAT antibodies were both used. As shown in Figure 3, the SMI-32 and CHAT stainings showed cell bodies, dendrites, and large axons. On the 10th day, as signs for maturation and high differentiation, the cultured SMNs developed large cell bodies with long neurites, which formed interconnected neurite networks and showed prominent arborization. The purity of SMNs was calculated by motor neuron marker (CHAT and SMI-32) immunofluorescence and DAPI staining. The results showed that the proportion of SMNs reached over 95%, which can meet the requirements of subsequent experiments.

### Morphological Changes of Primary SMNs Under OGD at Different Time Points

**Figure 4:**
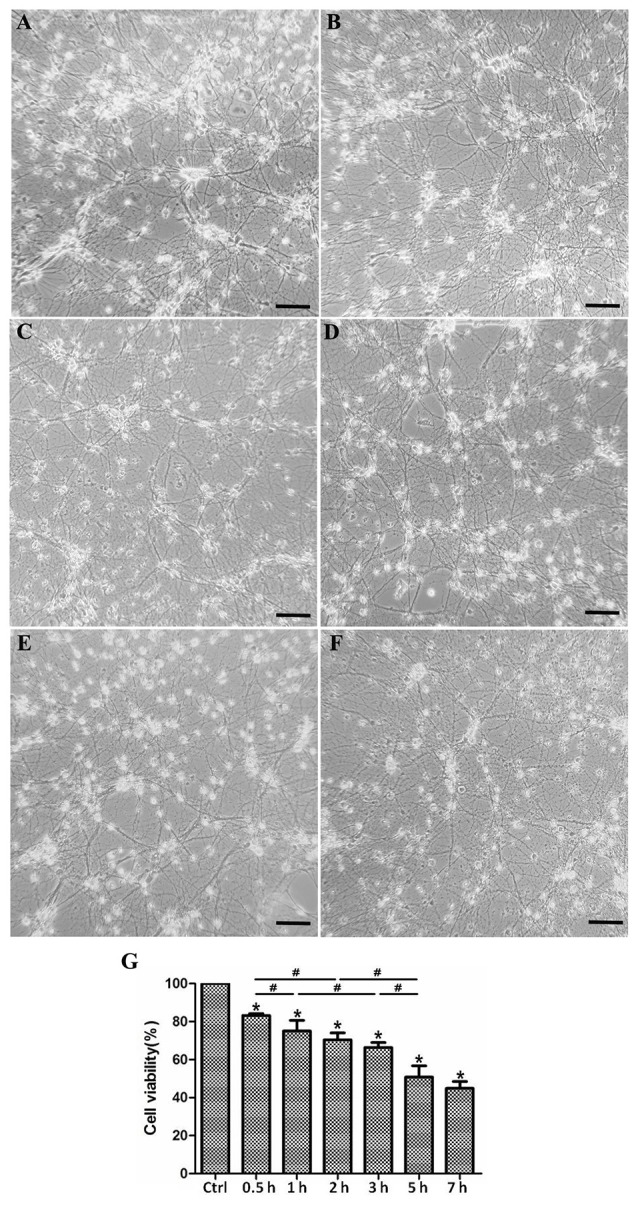
Morphological changes and cell viability of cultured SMNs under OGD for different time. Primary SMNs were cultured normally **(A)** or subjected to OGD for various periods of time (**B**: 1 h, **C**: 3 h, **D**: 5 h, **E**: 7 h, or **F**: 24 h, respectively), and the representative phase photomicrographs showed the morphologic changes (×250). **(A–F)** OGD resulted in destruction of neural networks, and the severity increased with the prolonging of OGD time. **(G)** Cell viability was reduced under OGD in a time-dependent manner. The cell viability was assessed using the CCK8 kit, and results were expressed as the percentage of CCK8 reduction. The absorbance of control cells was set at 100%. Data were presented as mean ± SD (*n* = 5 independent experiments). Statistical comparisons were carried out with one-way analysis of variance (ANOVA). *p < 0.05 compared with control group. ^#^*p* < 0.05 between groups.

OGD-induced SMN injury acts as a model to mimic spinal cord ischemia injury *in vitro* and results in neuronal insult. In this experiment, primary SMNs were cultured under OGD condition for 1, 2, 3, 5, 7, and 24 h, with motor neurons cultured in normal media and normoxia serving as control. As shown in Figures 4A–F, it seems that cells showed no obvious morphological changes within 1 h; however, most SMNs exhibited atrophic cyton and disrupted neurofilament after 3 h, and the injury continued to aggravate as time prolonged, with apparent impaired neural networks after 7 h and even massive cell loss for 24 h.

### Cell Viability Is Reduced Under OGD in a Time-Dependent Manner

We assessed the survival rate of SMNs after OGD insult. It was reduced under OGD in a time-dependent manner. According to Figure 4G, the cell viability declined sharply when cells were initially subjected to OGD (1 h), with significant changes every half an hour (control vs. 0.5 h, 0.5 vs. 1 h), but later declined relative slowly within 3 h (without significant change 1 vs. 2 h, and 2 vs. 3 h). Until 5 h upon OGD stress, viability declined markedly compared with 3 h. As time prolonged, cell viability declined more slowly, reaching a nearly 50% viability decrease in 7 h, but with no significant difference with 5 h.

### OGD Treatment Enhances Autophagy in Primary SMNs, and Autophagy Reaches a Peak at 5 h

**Figure 5:**
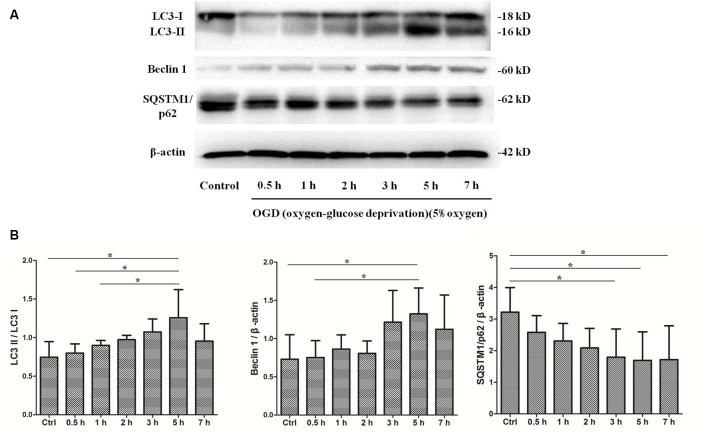
OGD treatment induces autophagy activation. SMNs were exposed to OGD for and indicated time, and the expression of light chain 3 (LC3) I, LC3 II, Beclin1, and SQSTM1/p62 were detected by Western blot **(A)**. **(B)** The quantitative results are shown after normalizing with β-actin as an internal control. Data were presented as mean ± SD (*n* = 3 independent experiments). Statistical comparisons were carried out with one-way ANOVA. *p < 0.05.

**Figure 6:**
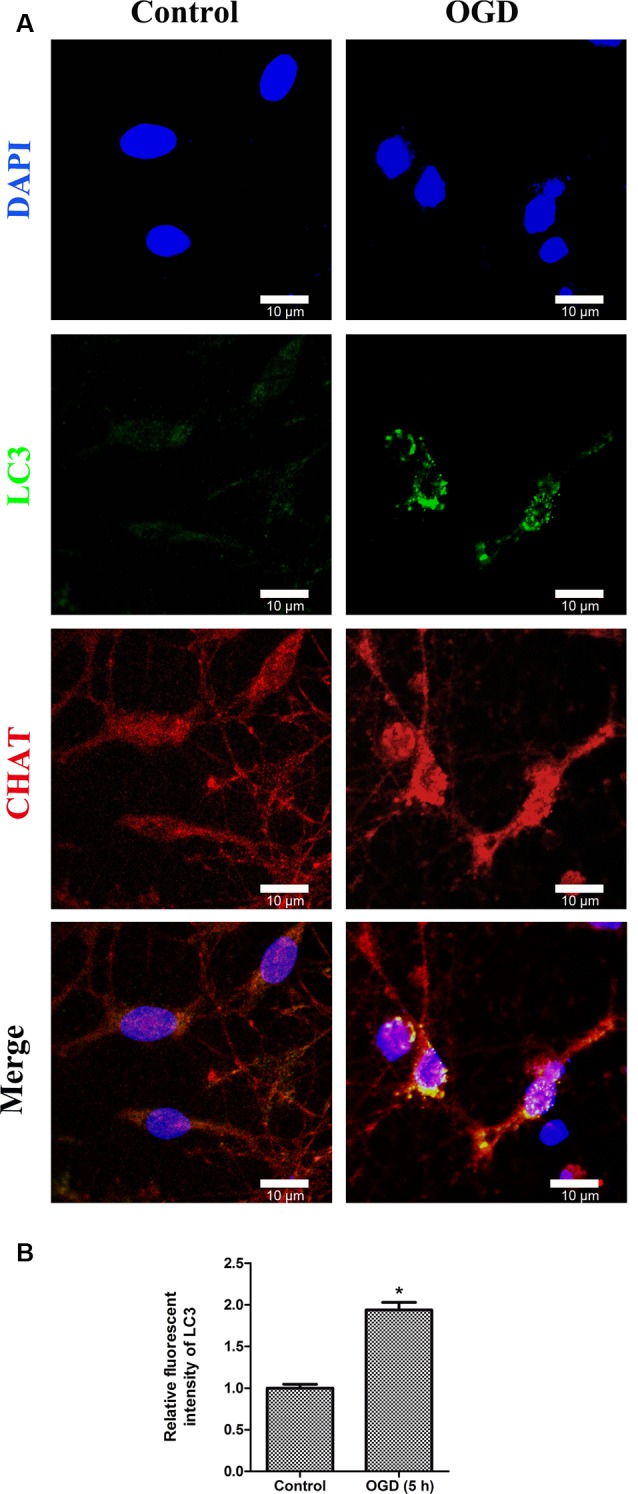
Double immunofluorescence stainings show the LC3-positive puncta in primary SMNs under OGD. **(A)** Representative fluorescent confocal images of cells counterstained with DAPI (blue, nuclei), autophagy-specific marker—LC3 (green) as well as motor neuron marker—CHAT (red) are shown (*n* = 3 independent experiments). Strong, punctate LC3 staining can be observed in primary SMNs under OGD. Scale bar presents 10 μm. **(B)** The relative fluorescent intensity of LC3 was analyzed using ImageJ. Data were presented as mean ± SD (*n* = 3 independent experiments). Statistical comparisons were carried out with unpaired Student’s *t*-test. *p < 0.05.

To determine whether autophagy was involved in SMNs under OGD conditions, we first examined the classical autophagy makers by Western blot assays. The microtubule-associated protein LC3, a mammalian homol of the yeast ATG8 gene (Aut7/Apg8), serves as the markers for autophagy. As shown in Figure 5, the bands showed the time course of autophagy induction under OGD, and the ratio of LC3-II/LC3-I was seen as a dramatic increase in SMNs after OGD for 3 h and reached a peak at 5 h. Immunofluorescence assay revealed strong and punctate LC3 staining in the OGD group (Figure 6), whose trends were consistent with that of Western blot analysis. Intriguingly, we can also observe that strong, punctate LC3 appeared much more in soma than in axons.

P62, also called sequestosome 1 (SQSTM1), is the selective cargo receptor for autophagy to degenerate misfolded proteins and serves as the marker for autophagic degradation. We detected the expression of SQSTM1/p62 and found that its level was diminished significantly after OGD for 3 h, which corresponded to the results of LC3. Additionally, Beclin1, the mammalian homolog of the yeast Agt6, forms a protein complex with class III phosphatidyl inositol-3 kinase within the autophagosome. Results showed that Beclin 1 expression was upregulated markedly and continually after OGD within 7 h.

### OGD Treatment Induces Autophagic Flux in Primary SMNs

**Figure 7:**
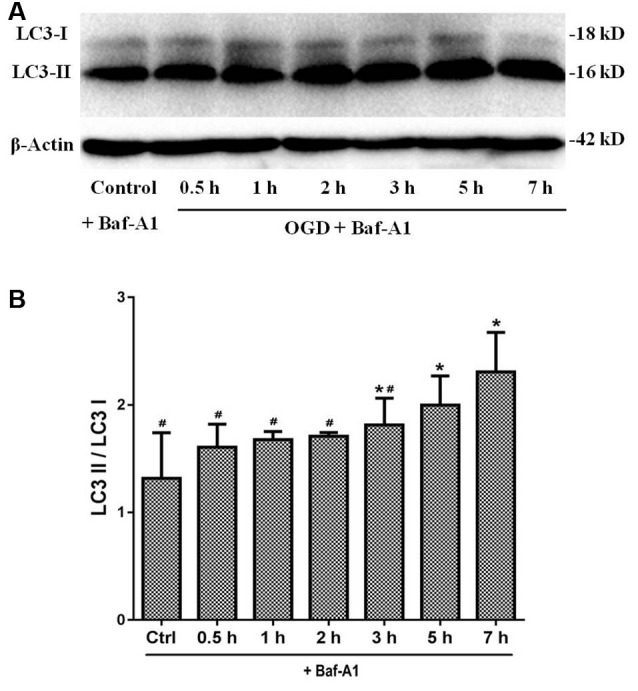
LC3 expression under OGD with Baf-A1 to observe the real state of autophagic flux. **(A)** The expression of LC3 I and LC3 II were detected by Western blot. In the presence of Baf-A1, LC3 II dramatically increased and accumulated. **(B)** The quantitative results are shown in the bar chart. Data were presented as mean ± SD (*n* = 3 independent experiments). Statistical comparisons were carried out with one-way ANOVA. *p < 0.05 compared with the control group. ^#^*p* < 0.05 compared with the 7-h group.

Given that autophagy was involved in primary SMNs upon OGD stress, we next decided to confirm that the enhancement of autophagy markers during the process was due to induction of autophagy or blockage of autophagosome maturation. For this purpose, lysosome inhibitor Bafilomycin A1 (Baf-A1) needs to be added in the culture medium to assess the responses of SMNs, which is a classical approach to observing the real state of autophagic flux. Baf A1 is a late-phase autophagy inhibitor, which acts by inhibiting vacuolar H + ATPase (V-ATPase) and thus prevents the maturation of autophagic vacuole fusion between autophagosomes and lysosomes (Gómez-Sánchez et al., 2015; Klionsky et al., 2016). Results in Figure 7 demonstrated that, in the presence of Baf-A1, LC3 II increased and accumulated, LC3-II/LC3-I displayed a much higher level when SMNs were treated with OGD plus Baf-A1 than OGD alone. This indicated that the increase in LC3-II levels by OGD was because of an increase in production rather than the decreased recycling of autophagy.

### Results of Transmission Electron Microscopy

**Figure 8:**
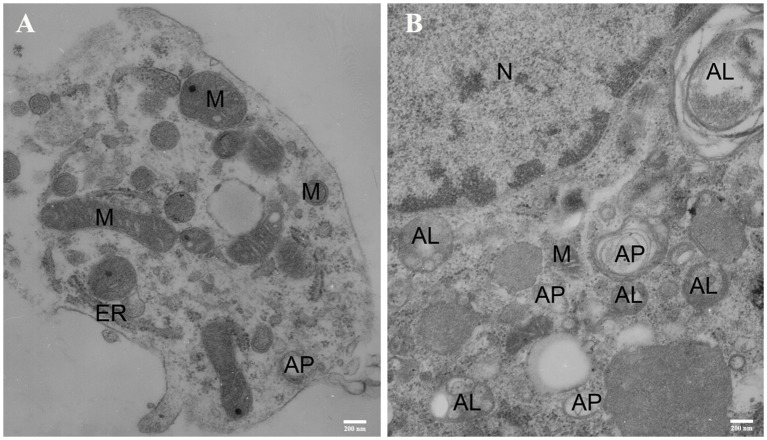
Representative electron micrographs (EM) of primary SMNs in the control **(A)** and OGD group **(B)**. The ultrastructural changes in primary SMNs after OGD injury are shown, and distinct autophagic structures are marked: autophagosome (AP), autolysosome (AL), mitochondria (M), endoplasmic reticulum (ER), cell nucleus (N). **(A)** The neuronal soma was plump and showed a normal morphology, with intact nuclear and plasma membranes. In addition, the mitochondria had a dense matrix and neatly aligned cristae, and no signs of autophagy were observed. **(B)** After OGD insults, crescent-shaped or goblet-like phagophores with double- or multiple-layer membrane structures and formation of autophagosomes can be observed, and autophagic vacuoles fused with lysosomes to form autolysosomes with single-layer membrane structures can also be seen. Images shown are representative examples from three independent experiments. Scale bars presents 200 nm.

The transmission electron microscopy (TEM) acts as one of the most important methods for monitoring autophagy (Swanlund et al., 2010). Autophagy was first detected by TEM in the 1950s, and it was originally observed as a focal degradation of cytoplasmic areas performed by lysosomes, which still remains the hallmarks of this process. Up until now, TEM is still the only tool that reveals the morphology of autophagic structures at a resolution in the nm range and shows the cellular substructure during the process, which allows the exact identification (Martinet et al., 2014). Therefore, we used TEM to examine the ultrastructural changes of SMNs subjected to OGD insult. In order to identify the typical differences, we chose the control cells and SMNs after 5 h of OGD. As shown in Figure 8, control cells contained normal-looking organelles, nucleus, and chromatin. The mitochondria had a dense matrix, neatly aligned cristae, and no signs of autophagy were observed. While after OGD insults, SMNs were found to contain many vesicles with typical morphological features of autophagosomes (AP). A number of double or multi-membrane structures, which engulfed cytoplasm fractions and organelles, can be observed in the cytoplasm. When autophagosomes fused with lysosomes, the inner membranes disappeared, and autophagosomes turned to be single-membrane autophagic vacuoles. The mitochondria displayed swelling with partially broken or dilated cristae. The TEM strongly once again suggested that overactivation of autophagy was triggered in primary SMNs subjected to OGD.

### Inhibition of Autophagy Exacerbates the Injury of Primary SMNs Exposed to OGD

**Figure 9:**
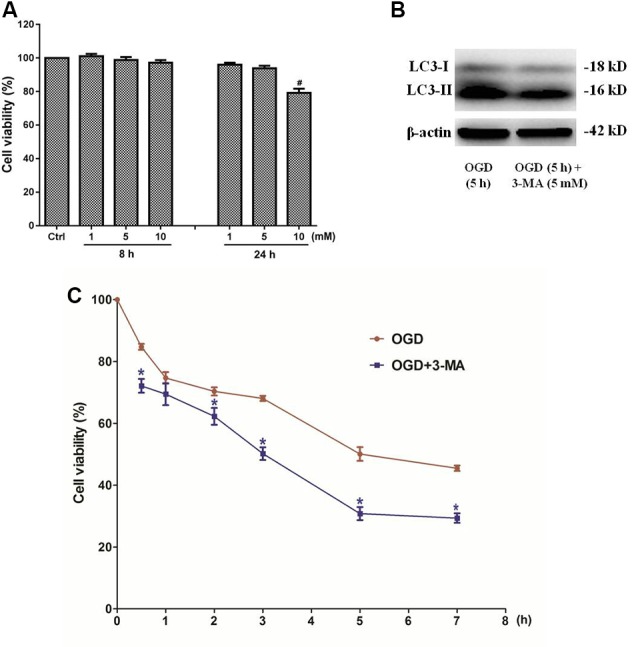
Inhibition of autophagy by 3-methyladenine (3-MA) exacerbates the injury of primary SMNs exposed to OGD. **(A)** Primary SMNs in normal conditions were treated with different dosages of 3-MA to select its safe dosage. **(B)** The autophagy inhibition effect of 5 mM 3-MA was confirmed by Western blot. To test it simple and clear, SMNs were subjected to OGD for 5 h in the presence or absence of 3-MA, and their expressions of LC3 I and LC3 II were detected. Results indicated that this dosage can significantly block autophagy in SMNs under OGD conditions. **(C)** The dosage 5 mM was applied in all the time points, and the cell viability of primary SMNs with or without 3-MA was assessed at different time points using the CCK8 kit. Results were expressed as the percentage of CCK8 reduction, and the absorbance of the control cells was set at 100%. Data were presented as mean ± SD (*n* = 5 independent experiments). Statistical comparisons were carried out with one-way ANOVA **(A)** or two-way ANOVA **(C)**. ^#^*p* < 0.05 compared with the control group. *p < 0.05 compared with cell viability in the same time point under OGD conditions.

In order to clarify the effects of autophagy in OGD-induced neuron injury, primary cultured SMNs were treated with autophagy inhibitor 3-MA, whose dosage has been selected to avoid cytotoxity (Figure 9A), and its corresponding inhibition effect was confirmed by Western blot (Figure 9B), followed by OGD for an indicated time, and then the cell viability was measured.

3-MA, a well-established inhibitor of autophagy, relatively selectively inhibits the class III phosphatidylinositol kinase (Wu et al., 2010), whose activity is required for autophagosome formation. It has been generally accepted and widely used as an inhibitor of autophagy. Our data showed that the cell viability was even decreased by 3-MA treatment (Figure 9C), indicating that inhibition of autophagy exacerbates the injury of primary SMNs exposed to OGD.

## Discussion

In the current study, we identified the dynamic time-course changes and role of autophagy in primary SMNs subjected to OGD. To the best of our knowledge, this report is among the first to clarify the autophagic expression changes in a primary cell model of spinal cord ischemia.

Primary cultures facilitate the limitation of similar *in situ* counterpart morphology and physiology. However, due to the low yields of motor neuron cultures from spinal cord before, hybrid cell lines of motor neurons (NSC 34 and VSC 4.1) have long been utilized and tacitly regarded as the most stable motor neuron cell line to mimic the pathophysiology of motor neuron disorders (Mosier et al., 1995; Eggett et al., 2000; Samantaray et al., 2006; Maier et al., 2013; Perera et al., 2018). Whereas, with the deepening and development of research, it has been found that there are obvious differences between the hybrid cell lines and primary cells, including the susceptibility of glutamate-induced death and calcium influx (Madji Hounoum et al., 2016). Hence, more and more scholars suggest that primary cells are more suitable as experimental models.

The isolation and culture of primary SMNs had been reported and described well (Schaffner et al., 1987; Martinou et al., 1989; Camu and Henderson, 1992, 1994; Graber and Harris, 2013); however, scarcely rapid and easy protocols have been achieved, let alone cellular models of SMNs. Previous literature mainly focuses on the possibility of successful isolation and culture of SMNs, by density gradient centrifugation, fluorescence-activated cell sorting or immunopanning, or a combination of these methods. Their results are inspiring, but the processes are often complicated, time-consuming, and hard to obtain sufficient quantities of SMNs once, which make it impossible to perform protein or RNA profiling systematically. Rapid and easy protocols facilitate mechanism studies or drug screens by performing downstream analyses (protein or RNA profiling), which require enough quantities of motor neurons *in vitro*. Therefore, we developed rapid, efficient, and reproducible procedures for the dissection of spinal cords and harvest of SMNs with high survival and purity (Chen et al., 2018), and develop cellular models of SMNs, which allow performing mechanism studies or drug screens.

Autophagy is an indispensable catabolic process in cell survival, differentiation, development, and homeostasis (Levine and Kroemer, 2008; Mizushima and Komatsu, 2011). The dual role of autophagy varies, either to protect cells through adaptive regulation or induce “autophagic cell death.” Accumulating studies have confirmed that autophagy activation is involved in the central nervous system injury and some neurodegenerative diseases (Tan et al., 2014; Martinez-Vicente, 2015; Guo et al., 2018), yet researches on autophagy in spinal cord injury are far from enough. Meanwhile, it is still debatable whether autophagy exerts a protective or destructive role in spinal cord injury. For instance, there are studies documenting that autophagy plays a protective role in spinal ischemia *via* sustaining autophagy (Fan et al., 2014). However, transient spinal cord ischemia can induce autophagy in motor neurons, which may result in neuronal death (Baba et al., 2009; Fujita et al., 2015). Meanwhile, even some research provided evidence that autophagy exerts opposing impact on early and later stages after spinal I/R injury: early activated autophagy alleviates spinal cord I/R injury by inhibiting apoptosis and inflammation, while later, excessively enhanced autophagy aggravates the injury (Fang et al., 2016). Nonetheless, the abovementioned researches are mainly *in vivo* studies, which did not show the concrete change of a certain cell type during the process of spinal cord ischemia injury, which we have tried to investigate in our studies.

Lack or deprivation of energy, such as oxygen or glucose, may lead to low physiological function, abnormal metabolism, and even cell death. In our experiment, the cell viability was reduced under OGD (Figure 4). According to the cell viability curve (Figure 4G), we clearly found that the cell viability declined sharply when cells were initially subjected to OGD, but later declined relatively slowly. Correspondingly, we detected the autophagic expression changes during the process (Figure 5). Concomitant with the rate of viability changes, it was interesting to find that autophagy was upregulated and kept increasing in the first few hours, reaching a peak at 5 h, suggesting that cells started to maintain the balance of internal environment after receiving adverse stimulus. However, since the OGD insult existed persistently, the cell viability continued to decline. Morphologically (Figures 4A–F), the SMNs showed slight injury in the first few hours and apparent impaired neural networks after 7 h, indicating that the normal physiological functions were unable to be maintained after stimulated by persistent adverse factors.

The microtubule-associated protein LC3, a mammalian homolog of the yeast ATG8 gene (Aut7/Apg8), takes an important part in the formation of autophagic vacuoles (Klionsky et al., 2016). The cytoplasmic form LC3 (LC3-I) is distributed throughout the cytoplasm diffusely and uniformly, but during autophagy activation, it will be conjugated to phosphatidylethanolamine to form LC3–phosphatidylethanolamine conjugate (LC3-II) and recruited to autophagosomal membranes (Mizushima and Yoshimori, 2007). When associated with autophagosomes, LC3 exhibits a typically apparent mobility in electrophoresis, changing from 18 kD to 16 kD and indicated as “LC3 puncta processing,” which is commonly referred to as LC3-II. Autophagosomes fuse with lysosomes to form autolysosomes, and intra-autophagosomal components are degraded by lysosomal hydrolases. At the same time, LC3-II in autolysosomal lumen is degraded. Therefore, lysosomal turnover of the autophagosomal marker LC3-II can reflect the autophagic activity to some extent, and detecting LC3 by immunoblotting and immunofluorescence have become reliable methods for monitoring autophagy and autophagy-related processes. The level of autophagy can be assessed by the ratio of LC3-II/I by immunoblotting and positive puncta of LC3 by immunofluorescence. In our study, the Western blot analysis revealed a significantly increase in LC3 level (Figure 5), which was further corroborated by immunofluorescence assays (Figure 6) and TEM (Figure 8). Meanwhile, the marker of autophagic degradation (SQSTM1/p62) diminished significantly after OGD for 3 h, and Beclin 1 expression was upregulated markedly and continually after OGD within 7 h (Figure 5).

Besides the fact that immunofluorescence assays confirmed the induction of autophagy during OGD, intriguingly, from the immunofluorescence images, strong LC3 puncta were assembly visualized in soma rather than in axons (Figure 6). Previous studies have demonstrated that retrograde transportation along axons back toward the neuronal cell bodies are critical during the autophagic process of neurons (Maday et al., 2012; Maday and Holzbaur, 2014; Zheng et al., 2019). It has already been proven that the process of retrograde transport along axons back toward the neuronal soma occurs in hippocampal, DRG, and cortical neurons (Lee et al., 2011; Maday et al., 2012; Maday and Holzbaur, 2014; Zheng et al., 2019). Our findings may provide some support for this theory in SMNs.

Detecting LC3 by immunoblotting and immunofluorescence are the basic ways to monitor autophagy and autophagy-related processes; however, these results can hardly represent the real state of autophagic flux since autophagy is a dynamic process with multiple steps. In this regard, lysosome inhibitor Baf-A1 was added to the culture medium to assess the responses of SMNs, which is a classical approach in observing the real state of autophagic flux. Actually, up until now, besides the lysosomal or vacuolar degradation inhibitor present or not in Western blot analysis, an alternative way to detect the autophagic flux is to measure the GFP–LC3 fusion protein with the aid of the GFP–LC3 expressing plasmid. In our experiment, we have tried both of the methods, but limited to the low transfection efficiency of GFP–LC3 expressing plasmid in primary SMNs, we only showed the results of autophagic flux detection by adding Baf-A1. In the presence of Baf-A1, LC3 II increased and accumulated (Figure 7), which indicated the increase in LC3-II levels by OGD owing to an increase in production rather than the decreased recycling of autophagy.

Later, in order to evaluate the role of autophagy, the autophagy inhibitor 3-MA was added in the culture system. It showed that blockade of autophagy activation by 3-MA aggravated cell injury, presenting worse cell viability.

## Conclusion

Taken together, by successfully establishing a primary cellular model of spinal cord ischemia *in vitro*, the dynamic time-course changes of autophagy and autophagy flux in SMNs was clearly investigated. Autophagy was upregulated in primary SMNs subjected to OGD, and autophagy reached a peak at 5 h. Besides, inhibition of autophagy exacerbated the injury of primary SMNs exposed to OGD, suggesting that autophagy plays a protective role during the process. Further studies need to explore the possible pathway and mechanisms underlying the effects of autophagy overactivation.

## Data Availability Statement

The datasets generated for this study are available on request to the corresponding author.

## Ethics Statement

The animal study was reviewed and approved by the Ethical Committee of Guangzhou University of Chinese Medicine.

## Author Contributions

SC and DLi conceived and designed the experiments. SC, RT, DLu, and ZX performed the experiments, analyzed the data, and wrote the manuscript. HL and DLi revised the manuscript. All authors have read and approved the manuscript for publication.

## Conflict of Interest

The authors declare that the research was conducted in the absence of any commercial or financial relationships that could be construed as a potential conflict of interest.
